# Systematic inference of indirect transcriptional regulation by protein kinases and phosphatases

**DOI:** 10.1371/journal.pcbi.1009414

**Published:** 2022-06-22

**Authors:** Christian Degnbol Madsen, Jotun Hein, Christopher T. Workman

**Affiliations:** 1 Department of Biotechnology and Biomedicine, Technical University of Denmark, Kongens Lyngby, Denmark; 2 Department of Statistics, University of Oxford, Oxford, United Kingdom; San Raffaele Hospital: IRCCS Ospedale San Raffaele, ITALY

## Abstract

Gene expression is controlled by pathways of regulatory factors often involving the activity of protein kinases on transcription factor proteins. Despite this well established mechanism, the number of well described pathways that include the regulatory role of protein kinases on transcription factors is surprisingly scarce in eukaryotes.

To address this, *PhosTF* was developed to infer functional regulatory interactions and pathways in both simulated and real biological networks, based on linear cyclic causal models with latent variables. *GeneNetWeaverPhos*, an extension of *GeneNetWeaver*, was developed to allow the simulation of perturbations in known networks that included the activity of protein kinases and phosphatases on gene regulation. Over 2000 genome-wide gene expression profiles, where the loss or gain of regulatory genes could be observed to perturb gene regulation, were then used to infer the existence of regulatory interactions, and their mode of regulation in the budding yeast *Saccharomyces cerevisiae*.

Despite the additional complexity, our inference performed comparably to the best methods that inferred transcription factor regulation assessed in the *DREAM4* challenge on similar simulated networks. Inference on integrated genome-scale data sets for yeast identified ∼ 8800 protein kinase/phosphatase-transcription factor interactions and ∼ 6500 interactions among protein kinases and/or phosphatases. Both types of regulatory predictions captured statistically significant numbers of known interactions of their type. Surprisingly, kinases and phosphatases regulated transcription factors by a negative mode or regulation (deactivation) in over 70% of the predictions.

## Author summary

In this work we addressed the challenging problem of inferring indirect (secondary) regulation by protein kinases and phosphatases via their activity on transcription factors. Although many protein kinase activity predictors have been developed for classes of protein kinases on specific amino acids within target sequences, our approach (PhosTF) provides predictions of regulatory activity for specific protein kinases and phosphatases on specific transcription factors. Our inference approach achieves this using the functional output observed in gene expression data of gene knock out strains, along with known transcription factor regulatory interactions. We formulated and tested a model for inference of regulation as well as a model for simulation of genes expression, transcription and translation. The simulation was used for computational validation of the inference method, which performed comparably to the best performers on a simpler inference problem in the DREAM4 competition. The inference method was then applied to yeast expression data, with significant validation by known kinase/phosphatase interactions. Over 15,000 novel regulatory interactions were predicted, suggesting that kinase activity provided a surprising level of repression of gene expression, either through the deactivation of activators or the activation of repressors.

## Introduction

Gene regulation is central to a cell’s ability to respond and adapt to changes in its environment. The control of transcription rates are directly regulated by transcription factors (TFs), and indirectly by chromatin state, cell signalling and other regulatory factors. Modulation of TF activity is often achieved through phosphorylation or dephosphorylation by protein kinases (PKs) or phosphatases (PPs), and TFs represent one of the most phosphorylated classes of proteins [1]. Direct or primary regulation by TFs can be mapped from protein-DNA binding experiments, e.g. by chromatin immunoprecipitation (ChIP) based methods, while evidence of indirect or secondary regulation by protein kinase and phosphatase can be observed from protein-protein binding as measured by yeast two-hybrid, or co-immunoprecipitation and mass spectrometry-based methods. These technologies suffer from false negatives due to the transient nature by which kinases and phosphatases bind their targets, as well as false positives [2]. Online databases containing protein interactions will sometimes report whether the data is collected from low- or high-throughput experiments, or whether they were observed reproducibly in multiple experiments, but information about data quality or functionality is often limited [3]. To infer functional regulatory interactions, one can draw from multiple sources of data, both protein binding data and evidence of regulation from mRNA transcript levels. In particular, when comparing the transcript levels from mutant strains, e.g. gene deletion (knock-out) or overexpression strains, to their background strains, the output of regulatory pathways can be observed by the resulting changes in mRNA levels. The loss or gain of a regulatory factor, e.g. a transcription factor or a protein kinase gene, will often generate altered transcript levels that imply functional regulation or a regulatory dependency between the perturbed regulator and the gene with an altered mRNA level [4].

Inference of functional regulation in TF-based regulatory networks were evaluated in the DREAM4 challenge [5]. A number of ground-truth *in silico* networks were used to generate knock-out, knock-down and wildtype gene expression levels that were provided to participants of the challenge. The ground-truth networks to be inferred were defined by 10 and 100 node adjacency matrices, originally constructed through sampling known TF regulatory interactions in model organisms. The provided gene expression levels were generated with the software GeneNetWeaver, which when given a ground-truth TF network, and a set of genetic perturbation, will apply differential equations to simulate mRNA and protein concentrations [6]. In this way, all regulators can be deleted or overexpressed (in silico) in turn and new steady-state mRNA output can be generated for each. However, GeneNetWeaver does not take into account phosphorylation or other post-translational modification that may result in secondary regulation. The focus of our approach was to extend the inference of TF-based regulatory networks to include the activity of such secondary regulators, in this case kinases and phosphatases, and to apply this method to the model budding yeast *Saccharomyces cerevisiae*, which has been extensively mapped for protein interactions. It should be noted that *S. cerevisiae* has primarily serine/threonine kinases and only limited tyrosine kinase activity. Although the vast majority of phosphorylations are of serine and threonine residues, yeast has tyrosine kinase *SWE1* and limited tyrosine kinase activity through the cross-activity of serine/threonine kinases *YAK1*, *KNS1*, and *HRR25*.

The majority of efforts to infer direct transcriptional regulation, often referred to as regulatory networks, have focused on TFs binding to promoter regions of their target genes. These networks are often modeled as directed acyclic graphs (DAG) of TF nodes interacting with nodes representing target genes. Applied in this biological context, each node value represents a protein’s concentration and each edge the direct regulatory effect, or activity, from node to node. Modelling regulation as a DAG has limitations on the inference accuracy considering that regulatory pathways often contain feedback (cycles) when target gene products are regulators themselves and, in turn, act further “upstream” in a regulatory pathway. The *linear cyclic causal models with latent variables* approach, otherwise known as Linear, Latent, Cycles (LLC), was specifically designed to address inference of causality in cyclic graphs [7]. It has been applied to infer TF regulatory networks in the DREAM4 challenge and was among the best performing approaches.

LLC describes a graph where node *i* has a value *x*_*i*_(*t*) at discreet time step *t* defined in [8] as

$$\begin{array}{c} x_{i}\left(t\right)=\sum_{j}b_{ij}x_{j}\left(t-1\right)+e_{i} \end{array}$$ (1)

where *b*_*ij*_ is the linear effect of node *j* onto node *i* and *e*_*i*_ is the latent term for node *i*. The equivalent expression in vector notation is shown in Eq (2a), and simplifies to Eq (2b) for *t* → ∞.

$$\begin{array}{ccc} x(t) & =Bx(t-1)+e & \left(2\text{a}\right)\\ x & =Bx+e & \left(2\text{b}\right) \end{array}$$

Perturbations to the system can be implemented by fixing the levels of specific regulators at a negative value in the case of a gene knock-out or a positive value for an overexpressed gene. Further details are described in subsection Intervention experiments of the Methods.

Methods have also been proposed for the inference of direct and indirect regulation that combine multiple likelihood functions for numerous types of evidence [3]. In such cases, maximum likelihood ratios can be calculated for each potential regulatory interaction (edge) by describing the likelihood ratios through factor graphs. Inferring indirect regulation by secondary regulators, such as protein kinases and phosphatases, is much more challenging since they do not regulate mRNA production rates directly, but rather modulate protein activity of other potential regulators. Although there have been many kinase prediction approaches (NetPhos, NetPhospan, PhosphoPredict) most have focused on the phosphorylation site prediction often without the ability to identify which kinase was likely responsible for the phosphorylation.

Recent studies utilizing mass spectrometry based proteomics or phosphoproteomics have investigated the activity of kinases and phosphatases in knockout studies [9][10]. In addition, approaches that infer regulation between human protein kinase (PK-substrate regulatory interactions) have recently revealed extensive circuits of kinases [11]. Their approach was based on an ensemble method combining multiple kinase-substrate scores and included phosphosite data for specific kinases or kinase families measured by phosphoproteomics, and gene expression data for quantifying co-expression and co-regulation. Although this method leveraged the combination of multiple data sources to achieve high performance, the method is limited to organisms where extensive kinase-phosphosite data is available.

**Fig 1:**
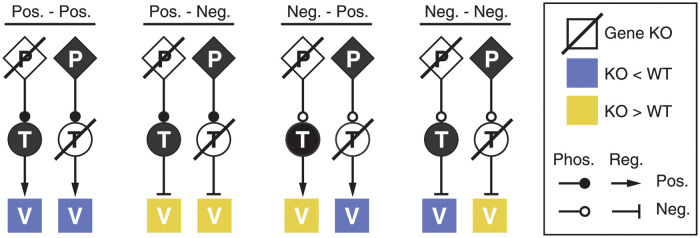
Effects of gene deletion on gene expression. Schematic of gene expression levels for a target gene ‘V’ relative to wildtype when a gene for either a secondary (*P*) or a primary regulator (*T*) is deleted. All combinations of positive (activating) and negative (repressing) regulation (Pos. or Neg. respectively) are shown. Activating or repressing phosphorylation (Phos.) are indicated with closed or open circles and regulation by TFs (Reg.) are indicated with pointed and flat arrowheads.

In this paper we have developed PhosTF, which builds on the LLC method. PhosTF can infer both direct regulation by primary regulators, represented by transcription factors, and indirect regulation by secondary regulators, represented in this study by protein kinases and phosphatases. Although secondary regulation by kinases and phosphatases is difficult to infer from any single knockout, their activities can be inferred in combination with TF knockouts as illustrated in Fig 1. We extended GeneNetWeaver simulations in GeneNetWeaverPhos to include regulation by phosphorylation, and describe a new method to infer gene regulatory networks based on a large compendium of knockout and overexpression transcription profiles, and an optional set of protein interactions, e.g. regulatory protein-DNA or protein-protein, as constraints.

## Results

The PhosTF method was developed to allow for the inference of direct and indirect regulation for a comprehensive set of primary regulators (*T*), potentially consisting all known transcription factors, and a large set of secondary regulators (*P*), in this case consisting of the known protein kinases and phosphatases. Although genes that affect secondary regulation through phosphorylation or de-phosphorylation were considered here, genes with other molecular functions that influence TF activity could also be included in this framework, e.g. ubiquitinases, acetylases, methylases, or sumoylases. The resulting method was capable of inferring regulation between secondary and primary regulators, and from primary regulators to their regulatory targets (*V*) (see Table 1 for full gene set definitions).

**Table 1:** Node set definitions.

Set	Role	Molecular function
PK	Secondary regulators	Protein kinases
PP	Secondary regulators	Protein phosphatases
*P*	Secondary regulators (PK ∪ PP)	Any PTM
*T*	Primary regulators	Transcription factors
*R*	All regulators (*P* ∪ *T*)	
*O*	Observed non-regulators	
*V*	All vertices (*R* ∪ *O*)	All

PhosTF was applied to both simulated and experimental data sets. Initially, a number of small- to medium-scale simulated data sets were used to test the inference performance, as these represented examples where the regulatory interactions were known. Subsequently, PhosTF was tested on a large compendium of experimental data collected for budding yeast, *S. cerevisiae* (see Table 2). An extensive set of regulatory interactions have been measured from transcription factors (primary regulators) to their regulatory targets in this model organism, 10^3^ − 10^4^ *d*(*T*, *V*) interactions, while only a very limited number of regulatory interactions are known between secondary and primary regulators, ∼ 10^2^ *d*(*P*, *T*) interactions.

**Table 2:** Yeast perturbation data resources.

Resource	Source	Tech.	Genes	Exp.
PK & PP KO	Holstege *et al.* [15]	DNA-MA	6109	163
KO	Holstege *et al.* [16]	DNA-MA	6170	1484
PK & PP KO	Zelezniak *et al.* [10]	SWATH-MS	726	352 (97)
PK & PP KO	Fiedler *et al.* [17]	DNA-MA	6184	2
PK & PP KO	Hu *et al.* [18]	DNA-MA	6253	5
TF KO	Hu *et al.* [18]	DNA-MA	6253	264
TF KO	Chua *et al.* [4]	DNA-MA	6222	102
TF OE	Chua *et al.* [4]	DNA-MA	6222	110

### Inference on simulated networks

**Fig 2:**
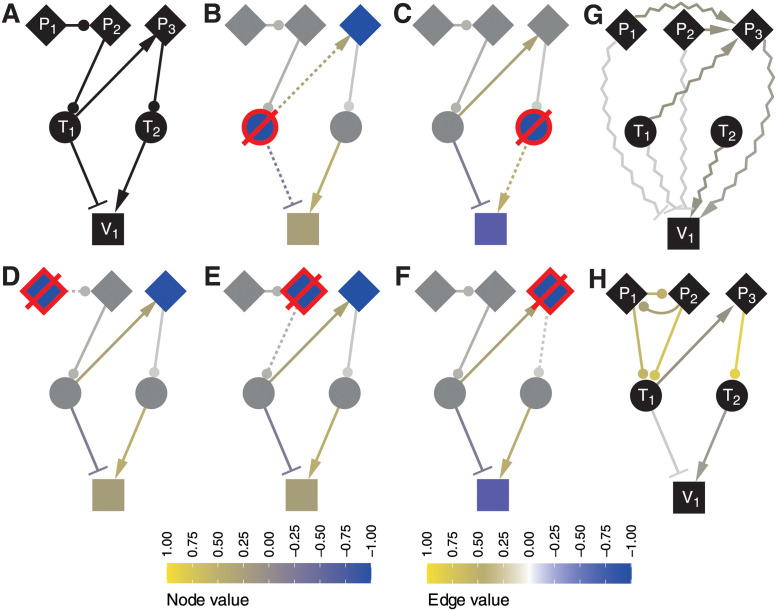
Small example network with unresolvable ambiguity. The true network (A), KPs (diamonds), TFs (circles) and target gene ‘V_1_’. The resulting mRNA outputs from simulated KO experiments are shown in (B-F) and represents the data used for inference. A graph representing the cumulative (total) influence through all pathways is shown in (G), and the inferred regulatory interactions are shown in (H). The node value color scale applies to (B-F) where colors show mRNA log fold-change values for each knockout. The Edge value color scale shows the “true” regulatory weights in (B-F) and the inferred values in (H). The knockout protein is indicated with a red border and strike-through. Dotted edges indicate the direct effects that were removed by the knockout.

Validation of PhosTF was performed on constructed regulatory networks and their simulated output in order to allow for inference where the regulation was known and to allow for the assessment of performance. Inference settings, such as the regularization strength λ was decided from tests on small archetypal graphs, shown in Figs 1 and 2. λ was tested with values 10, 1, 0.1, 0.01, and 0, where λ = 1 and λ = 0.1 were both found to fully recover the true network (see Regularization strength on 4 type example in S1 Text). This resulted in inference settings with λ = 0.1, that were then applied to 10 networks modified from the DREAM4 challenge to include secondary regulation.

Fig 2A shows a constructed 6-node network along with the resulting simulated levels in the in silico knock-out of each regulator (Fig 2B–2F). The way in which regulation is inferred is illustrated in the total effects graph in Fig 2G. The total effects concept is taken from Hyttinen *et al.* 2012 [8] where a total effect *t*(*x_i_* ⇝ *x_j_*) from node *x*_*i*_ onto node *x*_*j*_ is defined as the sum of all paths from *x*_*i*_ to *x*_*j*_. If there is only a single sample of each knockout experiment, then the total effect from node *i* to *j* is simply $t\left(x_{i}⇝x_{j}\right)=x_{j}^{\left(i\right)}/x_{i}^{\left(i\right)}$ where superscript indicates the knockout. The log fold-changes shown in Fig 2B–2F were calculated from simulated steady-state values (after convergence) of the ODE model defined in Eq (9) for the knockout and wildtype (background) strains.

As illustrated in Fig 2D and 2E, a situation can arise where the total effects from two secondary regulators (nodes *P*_1_ and *P*_2_) are very similar. This makes it difficult to infer the exact regulatory mechanism from perturbation data alone as shown in the inferred network Fig 2H. Due to the ambiguity of this particular challenging example, two additional edges were given non-zero weights. However this behaviour was modified by changing the regularization of *P* edges, or tuning the hyperparameter of the cost function (see Cost function in Methods).

### Performance on simulated networks

PhosTF performance was then assessed on 25 medium-scale simulated regulatory networks (see Network Construction for Simulation in Methods). These 100-node networks had on average: 20 *T*s and 20 *P*s, 13 *d*(*P*, *T*), 13 *d*(*P*, *P*), 25 *d*(*T*, *P*), 21 *d*(*T*, *T*), and 102 *d*(*T*, *O*), where *d*(*R*, *V*) denotes directed edges from regulator source *v*_*j*_ ∈ *R* to target *v*_*i*_ ∈ *V*, and where *O* is the set of non-regulating nodes. Both primary and secondary regulatory edges were inferred, i.e. *d*(*T*, *V*) and *d*(*P*, *R*). The only data given to PhosTF were the simulated log fold-change values and whether each node belonged to *P*, *T* or *O*.

**Fig 3:**
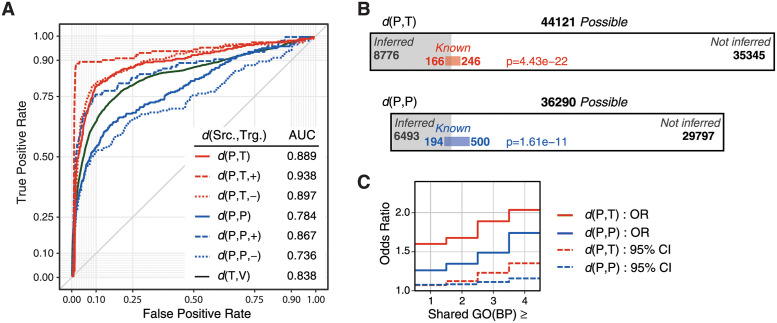
Performance evaluations. Edge inference assessment on simulated networks (A) with complete knowledge of regulatory interactions, and on yeast (B and C) where very limited examples were known. ROC curves illustrate performance for inferred edges pooled from all 25 simulated networks, each containing 100 nodes. Different types of regulation were assessed independently as indicated in the legend. Gene set ‘V’ refers to any gene. Area proportional diagrams for yeast inference are shown in (B). Gray areas represent the proportion of predicted interactions relative to the total possible for each type. Performance on the validation set (Known) are shown as colored areas representing the proportion of the known interactions that were predicted (or not predicted) for the two types of secondary regulation, *d*(*P*, *T*) in red and *d*(*P*, *P*) in blue. The counts of each interaction type are also shown next to each of the four areas in the two proportional diagrams. Fisher’s exact test *p*-values represent the chance of observing the prediction performance by random chance. Odds ratio for the number of shared GO Biological Process (BP) terms between interacting genes (C) based on the number of shared GO terms for inferred compared to uninferred edges. Dashed lines show the 95% confidence intervals of the odds ratios.

Results of a Receiver Operator Characteristic (ROC) analysis can be seen for the different types of regulatory interactions in Fig 3A. These curves show the trade off between sensitivity (True Positive Rate) and specificity (indicated by False Positive Rate) when selecting edges by the amplitude of the weights (|*w*_*ij*_|). Each ROC curve was generated on a merged list of potential edges from all 25 networks.

This analysis showed that regulation between secondary and primary regulators could be more accurately predicted than between two secondary regulators, and, surprisingly, more accurate than predictions from primary regulators (TFs) to their target genes. These curves were summarized for comparison by calculating the area under the ROC curves (AUC). It was not surprising that secondary regulation of TFs was easier to detect than regulation between two secondary regulators, since the latter are more distant in a regulatory pathway from the transcriptionally regulated genes. It is less clear why primary regulation achieved a lower AUC when compared to that for secondary regulation *d*(*P*, *T*) in these networks.

Overall, these results demonstrated a high performances for all types of *d*(*P*, *T*) regulation where an AUC of 0.89 was achieved. Even higher performances were observed for positive (*d*(*P*, *T*, +), AUC = 0.94) and negative regulation (*d*(*P*, *T*, −), AUC = 0.90) when assessed separately. We observed similar prediction performance for primary regulation by transcription factors (*d*(*T*, *V*), AUC = 0.84) compared to the methods assessed in the DREAM4 challenge (TF regulation only), where LLC had an overall AUC = 0.76 [8], and an AUC = 0.83 was achieved for the best performance on the original 100-node networks by team “ALF” [12]. Unusually, the ROC analysis showed that secondary regulation could be inferred more accurately for positively regulating edges between two secondary regulators, *d*(*P*, *P*, +), when compared to negative regulation, *d*(*P*, *P*, −). A differences in prediction performance was also observed between positive and negative modes of *P* regulation of transcription factors, again where positive regulation was predicted more accurately.

### Inference performance on yeast data

Regulatory network inference for yeast was applied to a large curated set of yeast gene expression studies (transcription profiles) for gene knockout and overexpression strains. Direct measurements of phosphorylation sites on regulators, and other prior knowledge were used to define validation sets for secondary regulation. The binding of transcription factors to DNA (see Yeast Data in Methods) and other evidence for direct transcriptional regulation by transcription factors was used to define the set of regulons, i.e. a TF and its regulatory targets. While the regulation from transcription factors is well studied in *S. cerevisiae* (>20,000 interactions), the number of known protein kinase and phosphatase regulatory interactions on transcription factors, or between such *P* proteins is very small (412 and 694 interactions respectively).

In an effort to focus on the inference of regulation between secondary and primary regulators, regulatory interactions were only inferred from *P* regulators to either other *P* or *T* regulators. For this reason, only weights for the known *T* regulatory interactions were estimated, thus reducing the complexity of the inference problem. By contrast, all possible *d*(*P*, *T*) and *d*(*P*, *P*) edges were included in the inference. Edge weights were trained (see Network Construction in Methods) and then separately filtered for *d*(*P*, *T*) and *d*(*P*, *P*) edges with a false discovery rate (FDR) threshold *q* < 0.05 resulting in ∼80 substrates per *P*, which was comparable to the average number of kinase targets previously reported (47) [1]. In total, 8776 *d*(*P*, *T*) and 6493 *d*(*P*, *P*) were predicted at this FDR threshold for 146 protein kinases and 45 protein phosphatases.

The two types of inferred *P* edges were evaluated relative to the limited set of known interactions (Fig 3B) using Fisher’s exact test. Despite the small size of the validation set (412 *d*(*P*, *T*) and 694 *d*(*P*, *P*) interactions), representing only 1% of the possible secondary regulatory interactions, PhosTF predicted 40% of the known *d*(*P*, *T*) and 28% of known *d*(*P*, *P*) interactions, which was highly significant (*p* < 10^−10^) when compared to the rate of predictions overall (20% and 18% respectively). These results were compared to predictions made by the protein sequence based substrate prediction method NetPhorest [13]. NetPhorest included 33 protein kinases which could be found in the evaluation set (where the source node is among the 33). The top scoring NetPhorest edges were selected in a number proportional to the number of inferred edges shown in Fig 3B and resulted in Fisher’s exact test *p* < 0.05 for both *d*(*P*, *T*) and *d*(*P*, *P*) NetPhorest predictions. The NetPhorest predictions contained 30% of the possible known secondary regulatory interactions with transcription factors compared to the 40% captured by PhosTF.

The regulatory interactions inferred by PhosTF were also evaluated with respect to shared Gene Ontology (GO) terms between regulator-target pairs. Since each gene can be assigned multiple GO terms, any two genes can be assessed for similarity in biological processes or molecular functions by the number of GO terms they share in a particular ontology. The odds ratio of having one or more shared GO slim Biological Process terms (GO(BP)) for the source and target genes of an inferred edge (compared to an uninferred edge) were 1.52 and 1.20 for *d*(*P*, *T*) and *d*(*P*, *P*) respectively, see Fig 3C. This odds ratio was observed to increase with the minimum number of shared GO terms. For example, the odds ratio increased to 2.05 for *d*(*P*, *T*) and 1.67 for *d*(*P*, *P*) edges when source and target genes shared 4 or more GO terms. All odds ratio estimates were outside the standard 95% confidence interval (CI), which implied a biological significance to both types of predictions with respect to capturing regulatory relationships between proteins functioning together in known biological pathways.

**Fig 4:**
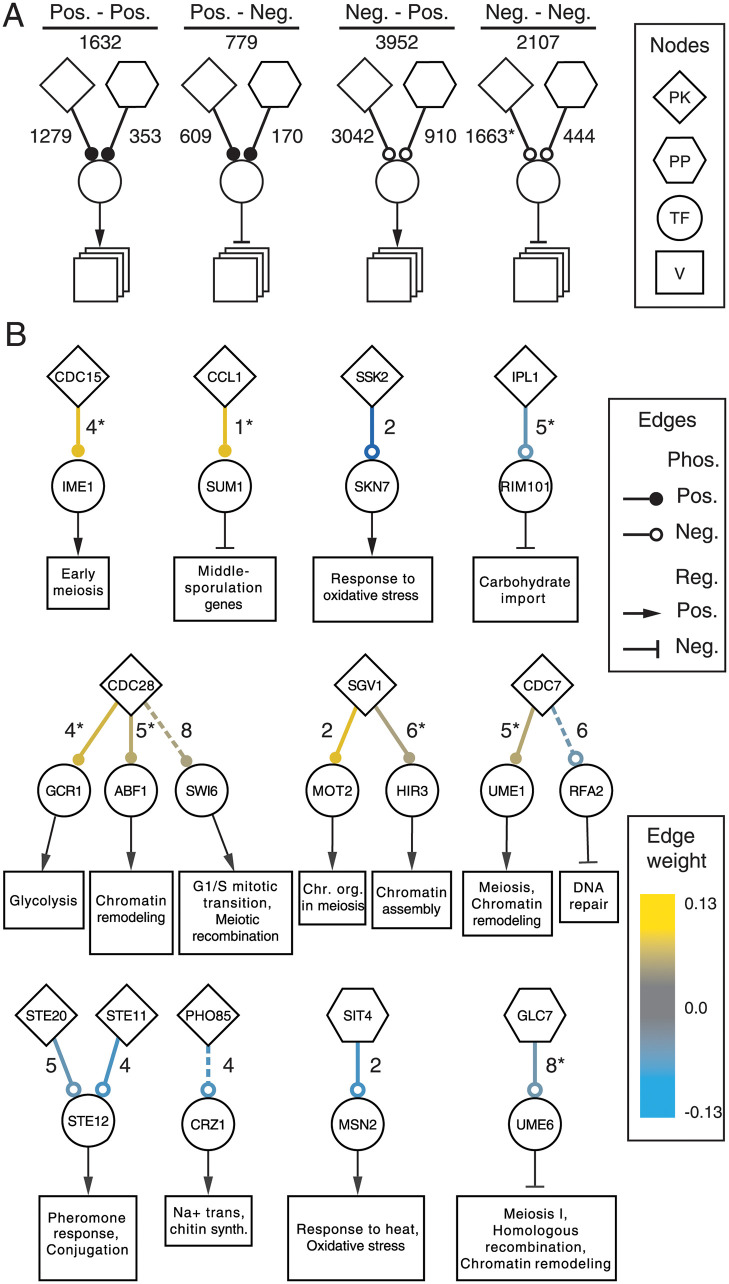
Inferred regulatory pathways. A summary of *d*(*P*, *T*) edges is shown in (A). Counts of inferred edges for each combination of regulation mode for *d*(PK, *T*) or *d*(PP, *T*) to either a primary transcriptional activator or repressor. Counts statistically larger than expected are marked with asterisk. The top scoring *d*(*P*, *T*) edges with shared GO terms are shown in (B). The number of GO terms shared between secondary regulators and TFs are shown next to each edge. A dashed line indicates the edges was present in the evaluation set, i.e. known interactions. An asterisk on the shared GO term indicates a prediction with no known evidence. Boxes represent regulons for the TFs and are labeled with representative significant biological process GO terms or the process the TF is known to regulate. The size of the box represents the number of genes in the regulon.

Due to the higher prediction performance of *d*(*P*, *T*) edges relative to *d*(*P*, *P*) edges, further analyses were performed for predicted regulatory interactions between secondary regulators and their targeted transcription factors. Counts for protein kinase (PK) and phosphatase (PP) edges were summarized in Fig 4A reflecting the combinations in Fig 1 for the positive or negative regulation of either a positively or negatively regulated TF-regulon. This simplified the role of a TF to have a single mode of regulation so as to focus on the role of *P* regulation and to avoid considering the combinations of paths from *P* to the TF and from the TF to its multiple targets. The proportions of these predicted regulatory interactions on TFs was tested relative to the expected counts in two *χ*^2^ tests (separately for *d*(PK, *T*) and *d*(PP, *T*)). The expected counts were calculated under the null hypothesis where *P* and *T* modes of regulation were independent. For instance, the expected number of positive *d*(PK, *T*) edges onto a TF activator (Pos.-Pos.) is the fraction of *d*(PK, *T*) edges that are positive × the fraction of activating *T*, scaled by the total number of *d*(PK, *T*) edges. It was found that negative PK-regulation of TFs that negatively regulate gene expression of their regulons (Neg.-Neg.) were over-represented by 21% (*p* < 10^−7^). The increased number of Neg.-Neg. pathways is contrasted with a relative under-representation of Pos.-Pos. pathways which were found to be 10% less than expected. The observed distribution of *d*(PP, *T*) edges was not observed to differ from the expectation based on a similar calculation.

The top positively and negatively regulated *P* pathways with shared GO terms were selected as candidates for further investigation and are shown in Fig 4B (see Methods). Of the resulting 16 *d*(*P*, *T*), three were known (in the validation set) and shared at least four GO(BP) terms. Of the remaining 13, five were found to be directly associated in the STRING database (combined score > 500), albeit only through types of evidence other than physical interactions. The remaining eight interactions (50%), indicated with an asterisk in Fig 4B, appear to be novel predictions.

## Methods

### PhosTF model definition

The inference is centered around a linear model of the influence nodes have on the values of other nodes, and how interventions on this graph can be used to infer the presence of edges in the graph, as well as their mode of regulation (positive or negative).

A number of node (vertex) sets were defined that represent the potential regulatory role of a gene, or its ability to be modeled by this regulation (Table 1).

#### Equilibrium equations

The following are the difference equations used to model the node attributes as a function of discreet time steps.

$$\begin{array}{ccc} x_{i}\left(t\right) & =\sum_{j\in T}w_{ij}a_{j}\left(t-1\right)+e_{i}^{\left(x\right)} & \left(3\text{a}\right)\\ y_{i}\left(t\right) & =\sum_{j\in P}w_{ij}a_{j}\left(t-1\right)+e_{i}^{\left(y\right)} & \left(3\text{b}\right) \end{array}$$

*x*_*i*_(*t*) represent the relative mRNA concentrations, specifically log_2_ fold-change mRNA concentration for a mutant relative to wildtype. The term *y*_*i*_ represents the relative regulatory activity of node *i*, and represents an extension of the LLC model when compare to Eq (1). In the context of this study, the unobserved variable *y*_*i*_ accounts for the effects of the phosphorylation state, but could in principle represent any post translational modification. Since phosphorylation can either activate or deactivate a regulator, it can be influenced by either kinases or phosphatases.

The edge value, *w*_*ij*_, defines the influence from node *j* to node *i* in a directed graph that may have cycles. As in the previous work, self-loops are avoided by enforcing *w*_*ii*_ = 0. *a*_*j*_(*t*) is a function of *x*_*j*_(*t*) and *y*_*j*_(*t*), which has to be defined in a meaningful way to combine the node concentration and activity attributes. $e_{i}^{\left(x\right)}$ and $e_{i}^{\left(y\right)}$ captures any latent concentration and activity contributions not explicitly mediated by the nodes in the network. In this study, *a*_*j*_(*t*) = *x*_*j*_(*t*)+ *y*_*j*_(*t*) (see Derivation of Inference Model in S1 Text), and the equilibrium equations simplify to the formulation in LLC if *a*_*j*_(*t*) = *x*_*j*_(*t*).

Eqs (3a and 3b) equivalently expressed in vector notation:

$$\begin{array}{ccc} x(t) & =W_{T}a\left(t-1\right)+e_{x} & \left(4\text{a}\right)\\ y(t) & =W_{P}a\left(t-1\right)+e_{y} & \left(4\text{b}\right) \end{array}$$

which for *t* → ∞ simplifies to (see S1 Text)

$$\begin{array}{c} x=Bx+e\;,\;B=W_{T}{\left(I-W_{P}\right)}^{-1} \end{array}$$ (5)

*W*_*T*_ = (*w*_*T*,*ij*_) and *W*_*P*_ = (*w*_*P*,*ij*_) are adjacency matrices containing *T* edges and *P* edges respectively. Since *T* ∩ *P* = ∅, then *W*_*T*_ and *W*_*P*_ can be formulated as a single weight matrix *W* with *W*_*T*_ = *WI*_*T*_ and *W*_*P*_ = (*I*_*T*_ + *I*_*P*_)*WI*_*P*_, where *I*_*T*_ and *I*_*P*_ are diagonal matrices with ones at indexes indicating nodes in *T* and *P*, and zero otherwise. It has been implemented as *W*_*T*_ = *W* ⊙ *M*_*T*_ and *W*_*P*_ = *W* ⊙ *M*_*P*_, where *M*_*T*_ and *M*_*P*_ are indicator matrices, and ⊙ is entry-wise multiplication.

Eq (5) represents the system at equilibrium without perturbation. The inference task is then to find a solution for *W*_*T*_ and *W*_*P*_ that satisfies the equality. There are many such solutions, which are narrowed down by considering the system under perturbation (see Intervention experiments). The solution space can also be reduced with information about known regulatory interactions. *M*_*T*_ was used to disallow certain TF edges (*d*(*T*, *V*)), those which lacked evidence, by setting the appropriate elements of *M*_*T*_ to zero. This approach was applied for the large inference performed on yeast data but was not used for the inference on simulated networks. Although equivalent operations could be applied to *M*_*P*_, insufficient information was available for this and was not performed.

#### Intervention experiments

For the purposes of this work, intervention experiments represented gene perturbations, where nodes in the model (representing genes and their gene products) are knocked out or over-expressed by changing their concentration parameter. In an experiment *k*, one or more nodes in $J_{k}$ (though typically one) are intervened on by setting

$$\begin{array}{c} {\left(x_{k}\right)}_{\left\{J_{k}\right\}}={\left(c_{k}\right)}_{\left\{J_{k}\right\}} \end{array}$$

where ***c***_*k*_ is a constant intervention, and this particular subscript notation indicates the subset of perturbed nodes (only). The combined expression for both intervened and passively observed nodes becomes:

$$\begin{array}{c} x_{k}=U_{k}Bx_{k}+U_{k}e_{k}+c_{k} \end{array}$$

***x***_*k*_ are node values for experiment *k* which were defined by a set $J_{k}$ containing indexes of intervened nodes. Multiple samples can be collected of each experiment *k*, however data was often limited to a single sample per gene. A knockout is not affected by transcription regulation so edges in *W*_*T*_ onto nodes in $J_{k}$ are removed by *U*_*k*_ = (*u*_*k*__*ij*_), which is a diagonal matrix with ones indicating passively observed nodes, and zeros indicating intervened nodes (*u*_*k*__*ii*_ = 0 for $i\in J_{k}$). The intervention term, ***c***_*k*_, contains zeros except for ${\left(c_{k}\right)}_{\left\{J_{k}\right\}}$ which are set to the log fold-change values measured in perturbation data. Again, ***e***_*k*_ represents noise and other latent effects.

#### Cost function

*W* can be inferred by minimizing ***e***_*k*_ for all experiments simultaneously with the *L*_2_-norm, i.e. a measure of the Sum of Squared Error (SSE):

$$\begin{array}{c} \text{SSE}={\left(∥{(I-B)X⊙U∥}_{2}\right)}^{2} \end{array}$$ (6)

where column *k* in *X* is ***x***_*k*_, column *k* in *U* is the diagonal of *U*_*k*_, and the norm is entry-wise. Minimizing SSE by itself will result in a non-parsimonious solution with many nonzero weights. Regularization approaches are used to generate fewer non-zero weights (induce weight sparsity) which is typically achieved by minimizing the *L*_1_-norm of the trainable weights, i.e. the entries (edges) in *W*. However, doing so assumes all primary and secondary regulation can be regularized identically. We instead regularize the (absolute) accumulated effects of weights defined as:

$$\begin{array}{c} B^{*}=W_{T}^{\prime }{\left(I-W_{P}^{\prime }\right)}^{-1} \end{array}$$ (7)

where $W_{T}^{\prime }$ and $W_{P}^{\prime }$ hold absolute elements of *W*_*T*_ and *W*_*P*_.

The intuition for this comes from first understanding that *B* is an adjacency matrix with entries identical to *W*_*T*_ for *d*(*T*, *V*) entries. For *d*(*P*, *R*) entries it holds the accumulated secondary regulatory effects. Regularization of *B* instead of *W* does not influence *d*(*T*, *V*) edges but only penalizes *d*(*P*, *R*) on their accumulated effects onto observable node values. However, accumulation of positive and negative influences through two separate cascades from a given node ∈ *P* onto a given node ∈ *R* can cancel out, leaving both cascades unrestricted. For this reason the absolute elements are taken of *W* resulting in B*.

The solution was then formulated as:

$$\begin{array}{c} \underset{W}{\text{arg min}}\;\text{SSE}+\lambda ∥{B^{*}∥}_{1} \end{array}$$ (8)

where the norm is entry-wise. All results were found using AdamW gradient descent [14] and regularization hyperparameter λ = 0.1.

### GeneNetWeaverPhos main equations

Data for benchmarking network inference was generated through numerical simulated with differential equations describing the concentrations of mRNA *r*_*i*_, protein *p*_*i*_ and activated protein *ψ*_*i*_ in a cell.

$$\begin{array}{ccc} \frac{\text{d}r_{i}}{\text{d}t} & =m_{i}^{\left(\text{RNA}\right)}f_{i}\left(\psi \right)-\lambda_{i}^{\left(\text{RNA}\right)}r_{i} & \left(9\text{a}\right)\\ \frac{\text{d}p_{i}}{\text{d}t} & =m_{i}^{\left(\text{Prot}\right)}r_{i}-\lambda_{i}^{\left(\text{Prot}\right)}p_{i} & \left(9\text{b}\right)\\ \frac{\text{d}\psi_{i}}{\text{d}t} & =(\sum_{j\in P}w_{ij}^{+}\psi_{j}+\lambda_{i}^{+})\left(p_{i}-\psi_{i}\right)\\ & -(\sum_{j\in P}w_{ij}^{-}\psi_{j}+\lambda_{i}^{-})\psi_{i} & \left(9\text{c}\right) \end{array}$$

*f*_*i*_ Eq (1) in S1 Text is a nonlinear function modelling transcription regulation taking into account TF binding cooperation and competition. It holds *f*_*i*_(***ψ***) ∈ [0, 1]. $m_{i}^{\left(\text{RNA}\right)}$ and $m_{i}^{\left(\text{Prot}\right)}$ are maximum transcription and translation rates. $\lambda_{i}^{\left(\text{RNA}\right)}$ and $\lambda_{i}^{\left(\text{Prot}\right)}$ are decay rates for mRNA and protein. $\lambda_{i}^{+}$ and $\lambda_{i}^{-}$ are the rate of passive activation and deactivation of protein *i*, i.e. not mediated by a specific regulator. $w_{ij}^{+}=\left|w_{ij}\right|$ if *w*_*ij*_ > 0, otherwise $w_{ij}^{+}=0$. Likewise, $w_{ij}^{-}=\left|w_{ij}\right|$ if *w*_*ij*_ < 0, otherwise $w_{ij}^{-}=0$.

Parallels can then be drawn between *r*_*i*_ here and *x*_*i*_ in the inference model (3a), however noting that *x*_*i*_ represents log fold-change mRNA concentration while *r*_*i*_ has to be simulated for both mutant and wildtype in silico networks before such value can be calculated from their comparison. Similarly, a parallel can be drawn between *ψ*_*i*_ here and *a*_*i*_ from the inference model, however GeneNetWeaverPhos and PhosTF are not designed to correspond to one another. Instead, the former serves simply as a method to generate artificial data and the latter to infer an underlying network from any log fold-change data.

#### Modeling of transcription regulation

For the simulations performed by GeneNetWeaverPhos, the proportion of maximum transcription was used to model the regulation of a gene. For gene *i*, the function *f*_*i*_(***ψ***) uses the amount of activated regulators to estimate this proportion given the regulatory inputs to gene *i* defined in the network. The way in which information from multiple regulators was integrated is described in detail in Modeling of transcription regulation in S1 Text. Regulator concentrations for each regulatory module were combined using a generalization of the Hill equation. In the special case of a module with a single regulator, the expression for *μ*_*m*_ from Eq (1) in S1 Text simplifies to a standard Hill equation for either an activator (10a) or a repressor (10b).

$$\begin{array}{ccc} \mu_{+}\left(\psi \right) & =\frac{\psi^{\nu }}{k^{\nu }+\psi^{\nu }} & \left(10\text{a}\right)\\ \mu_{-}\left(\psi \right) & =\frac{1}{1+{\left(\psi /k\right)}^{\nu }} & \left(10\text{b}\right) \end{array}$$

Here, *ψ* is the concentration of the transcriptional regulator which is able to bind the DNA, *k* is a dissociation constant, and *ν* is a parameter that shapes how binding sites respond to regulator saturation.

### Network construction for simulation

Five adjacency matrices from DREAM4 were each used 5 times to create 25 random adjacency matrices each with 100 nodes (see Generation of random adjacency matrices given to GeneNetWeaverPhos in S1 Text). Fully defined networks were then randomly generated with GeneNetWeaverPhos, which could subsequently be used to generate (simulated) log fold-change values. In the random networks, secondary regulators (protein kinases and phosphatases) were encoded as *P* which can both regulate positively and negatively.

TF regulons and their parameters were initialized by the same method as in GeneNetWeaver. If we define the decay rate from GeneNetWeaver as λ_decay_ and number of secondary regulatory edges onto node *i* as $\#w_{i}^{+}$ and $\#w_{i}^{-}$ for positive and negative regulation, then

$$\begin{array}{c} \begin{array}{cc} \lambda_{i}^{+} & ∼\{\begin{array}{cc} \lambda_{\text{decay}}, & \text{if}\;\#w_{i}^{+}=\#w_{i}^{-}=0\\ \lambda_{\text{decay}}\frac{\#w_{i}^{-}}{\#w_{i}^{+}+\#w_{i}^{-}}, & \text{otherwise} \end{array}\\ \lambda_{i}^{-} & ∼\{\begin{array}{cc} \lambda_{\text{decay}}, & \text{if}\;\#w_{i}^{+}=\#w_{i}^{-}=0\\ \lambda_{\text{decay}}\frac{\#w_{i}^{+}}{\#w_{i}^{+}+\#w_{i}^{-}}, & \text{otherwise} \end{array} \end{array} \end{array}$$ (11)

Edges $w_{ij}^{+}$ and $w_{ij}^{-}$ for *j* ∈ *P* were also sampled from the same distribution. Decay effects were cancelled by adding $\lambda_{i}^{-}$ and $\lambda_{i}^{+}$, respectively. Lastly, weights were normalized per target.

### Yeast data

Many types of data were collected for inferring a regulatory network for yeast and for evaluating the performance of said inference, in an effort to validate PhosTF.

#### Gene expression data

Experimental intervention data was represented by curated gene expression studies of genetic perturbations primarily consisted of gene knockouts and overexpression experiments, where (in most cases) a single gene was deleted or over-expressed (Table 2).

“Tech.” refers to the technology or type of experiment performed to obtain the relative expression data, either DNA microarray (“DNA-MA”), or Sequential Window Acquisition of All Theoretical Mass Spectra (“SWATH-MS”). All measurements were log_2_ fold-change mRNA expression levels for a mutant relative to wildtype, except for SWATH-MS data which were protein measurements instead of mRNA. For the data originally published by [18], values from the reanalysis by [19] were used. “Genes” is the number of measured genes for each experiment, and “Exp.” is the number of perturbation experiments characterized. The number of different mutated genes is given in parenthesis if different from the number of experiments (due to replicates). A total of 6395 different genes were measured over the 1306 experiments. Of these, 173 different secondary regulators (*P*) were genetically manipulated (knocked out or over expressed) in 828 experiments, and 272 different TFs (*T*) were similarly perturbed in 478 experiments.

#### Edge data

Experimental sources of *d*(*P*, *R*) edge data was used for evaluating inference performance. The evaluation data was identified from the union of *d*(*P*, *R*) edge data sets excluding NetPhorest, and filtered for substrates with a recorded phosphorylation site (see Node Sets in Network Construction). From STRING, validation interactions were only included where evidence that a kinase phosphorylated a target protein with a protein modification (PTMod) score > 250 were included. Other than PTMod, all other lines of evidence from STRING were ignored. YeastKID was filtered with threshold score > 4.52, corresponding to *p* < 0.05, which added > 400 *d*(*P*, *R*) interactions to the validation set. This resulted in validation sets of sizes |*d*(*P*, *T*)| = 412 and |*d*(*P*, *P*)| = 694.

Edge data for *d*(*T*, *V*) was used in *M*_*T*_ (see Equilibrium Equation in Methods) to define the primary regulation interactions used for the yeast inference problem.

“Value” displays the type of measurement if measurements were provided for the edges in the data set. Merged edge data was filtered by matching source and target nodes against mutated and measured genes from the perturbation data. “Entries” shows the number of measurements, and “Edges” is the number of edges after filtering by the sets *P*, *T*, and *V* (see Node Sets in Network Construction). Predictions scores were collected from NetPhorest using the provided reference yeast genome. The edge value “binding” refers to published binding evidence and “expression” refers to edges with evidence of expression regulation, where each edge only has evidence for positive or negative regulation. “Ambiguous regulation” refers to edges with evidence for both positive and negative regulation. “Score” and “scores” refer to single and multiple separate arbitrary scores for each edge measuring different types of interactions, notably a score for post-translational modification. Undirected interactions allowed for a potential *d*(*T*, *V*) edges in either direction.

The resulting *d*(*P*, *R*) set contained physical interaction data for 1106 of the 85371 potential edges (1.3%). Based on an integration of TF-binding data, a total of 21895 *d*(*T*, *V*) edges (7% of the 1467081 possible) were used in the yeast model inference. Briefly, extant data was found for 1258143 *d*(*T*, *V*) edges (86%), primarily from ChIP-seq or other TF-binding assays (see Table 3). If an edge was found multiple times in these data sets, the reported *p*-values from binding evidence were combined for each TF edge with Fisher’s method. For some data sets (e.g. Balaji *et al.* [24]) an overall *p*-value threshold was supplied but not individual edge *p*-values. In such a case the data-set threshold *p*-value was assigned to each edge in that data-set before applying Fisher’s method. Similarly, YEASTRACT binding data had a conservative *p* < 0.05 restriction enforced. A False Discovery Rate threshold *q* < 0.2 was used to filter the edges for combined significance resulting in final 21895 edges (∼95 per TF).

**Table 3:** Yeast edge data resources.

Resource	Source	Value	Entries	Edges
*d*(*P*, *R*)	BioGRID [20]		1433	279
*d*(*P*, *R*)	Fasolo *et al.* [21]		1025	59
*d*(*P*, *R*)	Parca *et al.* [22]		578	120
*d*(*P*, *R*)	Fiedler *et al.* [17]		667	267
*d*(*P*, *R*)	Ptacek *et al.* [1]		4290	341
*d*(*P*, *R*)	Yeast KID [23]	Score	31155	4364
*d*(*P*, *R*)	NetPhorest [13]	Prediction	220802	14058
*d*(*T*, *V*)	Balaji *et al.* [24]		12873	12716
*d*(*T*, *V*)	Beyer *et al.* [25]	*p*-value	13198	12707
*d*(*T*, *V*)	Lee *et al.* [26] [27]	*p*-value	2157385	1225212
*d*(*T*, *V*)	Horak *et al.* [28]	*p*-value	59359	51092
*d*(*T*, *V*)	YEASTRACT [29]	Binding	45206	43518
*d*(*T*, *V*)	YEASTRACT [29]	Expression	143344	138914
*d*(*T*, *V*)	YEASTRACT [29]	Ambiguous reg.	18304	18106
*d*(*V*, *V*)	STRING [30]	Scores	438768	
*d*(*P*, *R*)				2142
*d*(*T*, *V*)				2704
*V* − *V*	STRING [30]	Undirected score	1845966	
*T* − *V*				69808

BioGRID contained data for 40000 phosphorylation sites in 3918 proteins and another table with 111 kinases and 35 phosphatases mapped to 7561 of the sites. The BioGRID edge set was constructed through the mapping between the two tables.

#### GO data

Gene Ontology data was used for categorizing the *d*(*T*, *V*) mode of regulation, as well as assisting edge data in assigning proteins to sets *P*, *T*, and *O* (Table 4). GO Biological Process terms were curated for evaluation purposes.

**Table 4:** Gene ontology annotation resources.

Resource	Class	Entries	Proteins
TF activator	DNA-binding transcription activator activity, RNAP II-specific	68	48
TF activator	Positive regulation of transcription by RNAP II	309	223
TF activator	Positive regulation of transcription elongation from RNAP II promoter	63	46
TF repressor	DNA-binding transcription repressor activity, RNAP II-specific	38	23
TF repressor	Negative regulation of transcription by RNAP II	160	123
TF repressor	Negative regulation of transcription elongation from RNAP II promoter	4	2
PK	Protein kinase activity	256	137
PP	Protein phosphatase activity	58	45
Pathway	-	16107	6766

All GO resources were from AmiGO2 version 2020-01-01 [31] except for pathway resources retrieved from SGD [32]. The “Resource” column describes the interpretation of each GO term, and “Class” shows the filtered “GO class (direct)”. In the case of Biological Process GO terms, 100 different terms were possible to test. All AmiGO2 annotation queries were filtered by organism “Saccharomyces cerevisiae S288C”. “Entries” shows the number of entries for each query and “Proteins” shows the number of proteins with at least one entry.

Modes of regulation for primary regulators were classified according to curated GO evidence. GO evidence based on computational predictions alone was not considered in assigning regulatory modes. Low-throughput and direct experimental evidence was trusted over high-throughput and indirect evidence. This curation resulted in 191 TF activators, 65 TF repressors, 146 protein kinases, and 51 protein phosphatases.

### Yeast regulatory network construction

The data was processed to create an initial genome-scale regulatory network that was used for inference of secondary regulation. Subsections were ordered chronologically.

#### Definition of node sets

The *P* set was curated from the source nodes in *P* interaction data as well as the manipulated genes in *P* perturbation data (mRNA expression profiles). The *T* set was curated from the source nodes in TF interaction data filtered for target nodes in *V*, where one or more mRNA expression values were observed in perturbation data. *O* is the set of non-regulatory genes with at least one regulatory input from a primary regulatory (the subset of *V* not in *P* or *T*). Sizes of the distinct gene sets were |*P*| = 199, |*T*| = 231, and |*O*| = 5922.

#### Node values

Log_2_ fold-change (logFC) expression values were averaged across replicated perturbation experiments. This resulted in matrix *X* in Eq (6) consisting the logFC (or mean logFC), while *U* represented the mapping between manipulated and measured genes. Expression values for the specific genes that were knocked out (KO) or overexpressed (OE) were then adjusted by the following approach. The measurements of KO genes were adjusted by −4 logFC, which corresponded to an average KO gene level ∼100 times less than wildtype. Measurements of OE genes were adjusted by +1 logFC, corresponding to an average expression ∼4 times wildtype levels. In theory, a knocked out gene would have logFC of −∞, although using such values would not be feasible for inference. Empirical observations of knocked out genes were strongly influenced by cross-hybridization of other mRNAs and often resulted in only moderately negative logFC. For these reasons, the perturbation effects were enhanced (see Enhancing relative expression of genetically perturbed genes in S1 Text).

#### Initial d(T,V) weights

TF edge weights *w*_*ij*_ represent the relationship between the log fold-change value of a source node *v*_*j*_ ∈ *T* and target node *v*_*i*_ ∈ *V*. *w*_*ij*_ can be inferred from logFC values, but it is assumed that the physical binding evidence can adequately categorize TF-DNA interaction as present or absent.

TFs were categorized as either activators or repressors based on available data. The order of priority was: GO evidence, YEASTRACT and STRING combined with edge *p*-values, and lastly perturbation data. YEASTRACT and STRING described the mode of regulation for individual interactions. The *p*-values for either activating or repressing interactions were combined using Fisher’s method and compared for significance. Remaining unclassified TFs were categorized based on the average logFC of their targets in experiments where the TF was deleted, and if no such experiment existed, the classification was based on the sign of correlation between logFC values of the TF and its targets.

The mode of regulation for each edge was either assigned from the above listed sources, or inferred from the mode of regulation assigned to the TF. Edge weights were initialized as −1 or + 1 depending on mode of regulation. All other weights were set to zero and treated as invariant during training.

#### Initial d(P,R) weights

The variable (trainable) weights *w*_*ij*_ for *v*_*i*_ ∈ *R* and *v*_*j*_ ∈ *P* were initialized from a normal distribution with a small variance *σ*^2^ = 10^−4^. Initial *w*_*ij*_ for *v*_*i*_ ∈ *P* and *v*_*j*_ ∈ *P* were sampled randomly, however *w*_*ij*_ for *v*_*i*_ ∈ *T* were informed by Wilcoxon rank tests on *P* perturbation data. Absolute logFC values for each *P* with knockout data were compared for each TF with a one-sided Wilcoxon rank test. The tests compares absolute measurements from two groups of genes; the TF regulon and remaining genes. Significant *p*-values from these tests indicate which secondary regulators had influence on TF regulons. Instead of random sampling from a normal distribution, values were selected from a normal distribution for the quantile of the *p*-value. As a result, smaller *p*-values corresponds to larger |*w*_*ij*_|.

sgn(*w*_*ij*_) for *P*_*j*_ on *T*_*i*_ were initialized from equivalent two-sided Wilcoxon tests.

$$\begin{array}{c} \text{sgn}\left(w_{ij}\right)=-\text{sgn}\left(T_{i}\right)\cdot \text{sgn}\left({\hat{M}}_{ij}\right) \end{array}$$ (12)

sgn(*T*_*i*_) is the regulation mode of TF *i* curated from literature. ${\hat{M}}_{ij}$ is the estimated median of difference between the two groups.

#### Parameter estimation

Parameters were inferred with PhosTF for simulated data and yeast data alike. Initial states were described in Network Construction for Simulation and Network Yeast Regulatory Network Construction. Edge weights ($w_{ij}\in R$) were trained on simulated data from each 100-node network by minimizing Eq (8) for 15000 epochs. Only 50 epochs of gradient descent were performed in the case of training on the yeast data. Edge presence was scored as |*w*_*ij*_| and the sign was used for interpreting the mode of regulation.

### Evaluation of performance

#### Simulated regulatory networks

Performance for each inference was based on different edge weight thresholds, *θ*, where each Boolean classification generated a set of predicted present and absent edges. Prediction of an edge was either assessed as *w*_*ij*_ > *θ* for *d*(Sources, Targets, +), *w*_*ij*_ < −*θ* for *d*(Sources, Targets, −), or |*w*_*ij*_| > *θ* for *d*(Sources, Targets). When compared to the ‘true’ network edges (activating, repressing or absent), edge counts for true and false positives, and true and false negatives could be compiled. Comparing true positive rates to true negative rates, in an ROC analysis, allowed for the estimation of an area under the curve (AUC) as a measure of prediction performance.

#### Yeast regulatory networks

*d*(*T*, *V*) edges were constructed from binding data, so performance was not evaluated on the inferred edge weights for these edges. The evaluation of performance on *d*(*P*, *R*) was performed using experimental data since *P* edges were only inferred from perturbation data, as well as indirectly implicated through the restrictions applied to *d*(*T*, *V*) edges. Performance could furthermore be assessed using GO process terms since such data was also not used in the inference process.

Top scoring pathways were collected using thresholds of ≥ 1 to ≥ 6, where the source and target of *d*(*P*, *R*) edges shared GO slim biological process terms (“Pathway” in Table 4), are shown in Fig 4B. The 4 top *d*(*P*, *T*) edges (by *w*_*ij*_) were identified for each threshold: top two edges with highest and lowest edge weights (largest absolute edge weights for positive and negative regulation). The set of edges found for a particular shared GO term threshold often overlapped with those found for the other thresholds, resulting in the 16 shown.

## Discussion

A direct performance comparison for the inference of primary regulation showed that the performance of PhosTF on simulated 100-node networks was either comparable to or better than that of simpler simulation models applied in the DREAM challenges. Although, it was expected that primary regulation would be easier to infer than secondary regulation, we observed higher prediction performance for secondary regulation in these medium-sized simulated networks (AUC 0.9 for *d*(*P*, *T*) versus AUC 0.84 for *d*(*T*, *V*)). Considering this, PhosTF should be viewed as an advance due to the accurate prediction performance for secondary regulation in addition to state-of-the-art performance for prediction of primary regulation.

Inference of secondary regulation in yeast was significantly harder to perform and evaluate. Performance evaluation was challenged by the small size of the evaluation set compared to the set of potential edges, as well as the lack of a proper negative set. With so few known examples, it cannot be assumed that the majority of novel predictions were false positives, and that prediction specificities could not be realistically estimated. Sensitivity estimates were 0.40 for *d*(*P*, *T*) and 0.28 for *d*(*P*, *P*) predictions meaning that roughly 30–40% of what was known was inferred from this approach, see Fig 3. Nevertheless, the enrichment of known interactions in the prediction set was highly significant. Of the predicted 8610 new *d*(*P*, *T*) (secondary to primary) and 6299 new *d*(*P*, *P*) (secondary to secondary) regulatory interactions, 30–40% of each type would be expected to be real. PhosTF also outperformed the existing kinase specificity based predictions of NetPhorest and is sufficiently accurate to provide sets of predictions for further validation studies.

Approximately 70% of secondary regulatory interactions on transcription factors in yeast appeared to be negatively regulating (deactivating) their targeted transcription factors. This bias was observed to be even stronger for the predicted weights of the 166 *d*(*P*, *T*) edges that were in the validation set. In this case, 85% of the already known *d*(*P*, *T*) edges were estimated to have deactivating effects. Despite this surprising predominance of negative regulation by secondary regulators, the higher than expected prediction of de-repression pathways (Neg.-Neg.) suggests selection of indirect transcriptional activation by protein kinases through the negative regulation of repressors. When considering the net regulatory effects, the overall proportion of repressing pathways was 62% when summing Pos.-Neg. and Neg.-Pos. (Fig 1A).

We investigated whether the bias of negative secondary regulation was due to systematic aspects of the gene expression data for the various knock outs used for inference. Such biases could arise, for example, if large numbers of differentially expressed genes were non-specifically affected by different gene deletions. Importantly, non-specific deletion effects would only be expected to increase the two cases where secondary regulation of TFs was positive, i.e. Pos.-Pos. and Pos.-Neg. (Fig 1A), because these two type of pathways induce the same type of KO-affect on the targeted genes. Conversely, negative regulation of transcription factors requires that the transcriptionally regulated target genes change from up-regulation to down-regulation (or vice versa) between knock outs of secondary and primary regulators. Therefore, non-specific or consistent KO effects would only be expected to implicate positive secondary regulation. As a further check, the signs of differential expression for all perturbation measurements were compared between secondary and primary regulator perturbations for each predicted *d*(*P*, *T*) edge. The comparisons of signs across knockout profiles did not reveal systematic anti-correlation of transcriptional responses either, which additionally suggested that the negative secondary regulation bias was not expected by random chance.

One possible explanation for the over representation of negative secondary regulation could relate to the nucleocytoplasmic trafficking of TFs as a function of their phosphorylation state. Protein phosphorylation is known to regulate trafficking of proteins in and out of the nucleus, and is particularly relevant for TFs as this is where their primary mode of action takes place. The trafficking hypothesis would imply that phosphorylation more often facilitates retention of TFs in the cytoplasm, effectively deactivating them. This bias is not well supported by our current, albeit incomplete, knowledge of nucleocytoplasmic trafficking. There are as many or more anecdotal examples that describe phosphorylation promoting nuclear import than describe cytoplasmic retention [33]. Despite the inconclusive evidence, many examples are known where phosphorylation of TFs either facilitates cytoplasmic retention or nuclear export, e.g. Pho4p, Mig1p and Crz1p [34]. Considering that this bias for negative secondary regulation was also observed for phosphatases, nucleocytoplasmic trafficking alone will not be sufficient to explain this phenomenon.

Despite the relatively large number of regulatory interactions that could be predicted from our approach, a number of possible inference challenges were identified. Ambiguous solutions can arise even in simple network models using simulated perturbations. It was observed for inference on some small networks that if two secondary regulators were similar in their regulatory roles, it could become impossible to distinguish between one regulating the other, or both regulating the same target. The small example shown in Fig 2A, with 3 *P*_*i*_, 2 *T*_*j*_, 1 target gene *V*, and 6 regulatory interactions, illustrates an example where two secondary regulators have a similar role. Despite the ambiguity between *P*_1_ and *P*_2_, all 6 known regulatory interactions were correctly predicted along with two extraneous edges (false positives). These extra edges give *P*_1_ and *P*_2_ the same regulatory interactions in the network, both regulating each other and *T*_1_.

As PhosTF minimizes the cost function to provide a single inferred *W*_*T*_ and *W*_*P*_, it can in general be said that it provides a single parsimonious solution, with potential for random variation for repeated runs on complicated challenges. However, as presented in Fig 2 it is possible to balance regularization to let alternative inference pathways simultaneously appear.

Since ambiguous regulation or feedback cycles can result in prediction of false positive interactions, it was important to implement further regularization approaches in PhosTF to ensure sparsity in the inferred interaction network. To induce sparsity, approaches are typically applied to penalize edges, e.g. on *W*. From testing on small simulated networks, it was found that PhosTF performs much better when the regularization was applied to B* instead of *W*, where regularization is typically performed. This is likely because *L*_1_ regularization of *W* unevenly penalizes *d*(*P*, *P*) edges compared to *d*(*P*, *T*) and *d*(*T*, *R*) edges (*R* = *P* ∪ *T*). This improvement alone is likely why our prediction performance compares favorably to previous DREAM winners despite the additional challenges imposed by indirect regulation.

Other inference challenges could not be addressed by improved regularization approaches. For example, some regulatory effects can be silent due to the presence of compensatory pathways. Compensating signal transduction cascades are difficult to infer from perturbation data if only a single gene has been deleted from either cascade. This limitation can only be overcome with multiple knockouts in the same experiment. Environmental conditions may also prevent the observation of KO effects if secondary regulators are inactive under such conditions. This type of silent regulation can be observed in simulations when edge weights (activities) are initially set too close to zero. In these cases, the deactivation of a node with activity 0 will not be detected. Conversely, simulating the overexpression of a node with the maximum activity will also not be detected. Some steps were taken to avoid silent regulation in the simulations, e.g. setting the magnitudes lower for edges sharing the same target (see Generation of random adjacency matrices given to GeneNetWeaverPhos in S1 Text). Despite this, silent regulation present in experimental data cannot always be avoided, which means some regulation cannot be inferred. Extensions of the method may be required to better suit modeling of holoenzymes, as currently each node represents a single gene product. Such an extension could represent protein complexes as nodes in the network.

This study presented a novel regulatory inference method PhosTF as well as an extension of the GeneNetWeaver simulation tool, GeneNetWeaverPhos, which shows potential future use in inference approaches. Given enough computational resources, GeneNetWeaverPhos could be iteratively run with variations to the network structure, to minimize the difference between simulated and experimental gene expression levels. This could be accomplished with a Markov chain Monte Carlo approach such as the Metropolis-Hastings algorithm. PhosTF was demonstrated to infer secondary regulation in both small and large networks containing many hundreds of regulators. In addition to gaining a systems-wide understanding of how transcription factor activity is modulated by specific classes of secondary regulators (protein kinases and phosphatases), the inferred regulatory networks can be used to predict the effects of gene mutation, or the over- or underexpression of regulators. It is hoped that these extended regulatory networks can provide engineering targets for improved control of gene expression in bioprocess strains.

## Supporting information

S1 Text Method details. GeneNetWeaverPhos simulation and network construction details and derivation of inference model.
(PDF)

S1 Dataset Yeast interactions. Inferred *d*(*P*, *R*) edges and curated *d*(*T*, *V*) edges.
(TGZ)
