# Supplementary material for: Systematic inference of indirect transcriptional regulation by protein kinases and phosphatases
Source: PLoS Comput Biol. 2022 Jun 22;18(6):e1009414. doi: 10.1371/journal.pcbi.1009414 (PMC9255832; doi:10.1371/journal.pcbi.1009414)
Supplement: S1 Text — GeneNetWeaverPhos simulation and network construction details and derivation of inference model. (PDF) [file pcbi.1009414.s001.pdf]

Supporting Information:

Systematic inference of indirect transcriptional regulation by protein kinases and phosphatases

Christian Degnbol Madsen[1,2], Jotun Hein[2], Christopher T. Workman[1*]

1 Department of Biotechnology and Biomedicine, Technical University of Denmark, 2800 Kongens Lyngby, Denmark
2 Department of Statistics, University of Oxford, Oxford OX1, UK

Method details

Modeling of transcription regulation

Transcription regulation in GeneNetWeaverPhos is modeled by the proportion of maximum transcription (activation) for gene i as term f_i (1), which is used in Eq (9a) in the main text. A similar f_i term is mentioned in supplementary material of [1], and was originally developed on the basis of [2]. In GeneNetWeaver, f_i is a function of protein level, $\boldsymbol{p}$, however in GeneNetWeaverPhos it is a function of activated protein $\boldsymbol{\psi}$. f_i takes into account different types of transcriptional regulation, both regulator competition and cooperation for any combination of activators and repressors. A gene could be regulated as exemplified in Fig S1. The activation

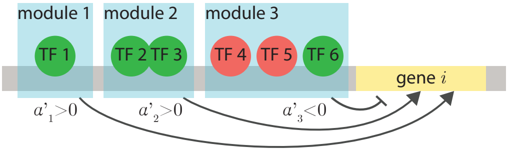

Fig S1. Example of transcriptional regulation in simulation. A gene regulated by $N = 3$ regulatory modules, where module 1 is regulated by a single activator TF, module 2 is bound by a complex of two TFs, which both have to be present for activation of the module, and activating and repressing TFs compete to bind the third module. Green: activation, red: repression.

level $f_i(\boldsymbol{\psi})$ of gene i is controlled by a number of regulatory modules. A (regulatory) module are groupings of TF regulators for which either competitive or cooperative binding dynamics are at play. A module may be thought of as a binding site. Each regulatory module m groups a number of TFs controlling its fraction of activation $\mu_m \in [0, 1]$. In this modeling framework there exist two discreet states for a module; either it is activated or inactivated, where μ_m can be considered the activated fraction over a short duration of time or the fraction of cells in a culture. From this we can realize that a given gene i will be in one of 2^N possible states, where N is the number of regulatory modules and a state is a unique combination of each module as either activated or inactivated.

$$
\begin{aligned}
f_i(\boldsymbol{\psi}) &= \sum_{s=1}^{2^N} \alpha_s P\{S = s|\boldsymbol{\psi}\} \\
\alpha_s &= \mathrm{clamp}\left(0, \alpha'_0 + \sum_{m\in\sigma_s} \alpha'_m, 1\right) \\
P\{S = s|\boldsymbol{\psi}\} &= \prod_{m\in\sigma_s} \mu_m \cdot \prod_{m\notin\sigma_s} (1-\mu_m) \\
\mu_m &= \begin{cases} \frac{\prod_{j\in A_m} \chi_j}{\prod_{j\in A_m\cup R_m} 1+\chi_j}, & \text{if } m \notin C \\ \frac{\prod_{j\in A_m} \chi_j}{1+\prod_{j\in A_m} \chi_j}, & \text{if } m \in C \\ & \wedge R_m = \emptyset \\ \frac{\prod_{j\in A_m} \chi_j}{1+\prod_{j\in A_m} \chi_j + \prod_{j\in A_m\cup R_m} \chi_j}, & \text{otherwise} \end{cases} \\
\chi_j &= \left(\frac{\psi_j}{k_j}\right)^{\nu_j}
\end{aligned}
\tag{1}
$$

$\boldsymbol{\psi}$ are nondimensionalized active protein concentrations. All parameters in f_i are specific to gene i, so subscript i is omitted here for clarity. α_s is the fraction of maximum gene transcription for state s. α'_0 is a baseline activation of gene i, i.e. the activation of gene i when none of its regulatory modules are active. α'_m is the relative activation of gene i by an active module m. A regulatory module can be repressing if $\alpha'_m < 0$. We constrain $\alpha_s \in [0, 1]$ by using $\mathrm{clamp}(l, x, u) = \max(l, \min(x, u))$. σ_s is the set of modules active for gene i in state s. $P\{S = s|\boldsymbol{\psi}\}$ is the probability that gene i is in state s given the current active protein concentrations $\boldsymbol{\psi}$, or equivalently the expected fraction of gene i in state s. $P\{S = s|\boldsymbol{\psi}\}$ is defined by the the probability that modules in σ_s are active and the rest are inactive. The probability that a module is active, is equivalent to μ_m; the expected fraction of active module m (given $\boldsymbol{\psi}$). μ_m is defined in three separate ways depending on whether the module is bound by a TF complex ($m \in C$), if some of the TFs that bind to module m are repressors ($\exists j \in R_m$) or if all are activators ($R_m = \emptyset$). The formulation for μ_m is a generalization of the well known Hill equations, which describe binding site activation as a function of a single regulator concentration. The formulation of μ_m simplifies to a Hill equation when there is only a single activator or a single repressor regulating gene i. k_j is a dissociation constant for TF j, and ν_j is the Hill coefficient, which represents the degree of cooperativity in binding, i.e. to which degree binding affinity will increase or decrease with the presence of bound regulator.

Generation of random adjacency matrices given to GeneNetWeaverPhos

Simulated data was created from a gene regulation network defined not only by nodes and edges, but also by specific regulation parameters (rate constants) and configurations of regulatory modules, see Eq (9) in the main text. Network constants such as decay rates and α'_m (1) were set randomly based on a graph with vertices $V = P \cup T \cup O$ and edges defined as an adjacency matrix D with elements $d_{ij} \in \{-1, 0, 1\}$. $d_{ij} = -1$ indicates repression if node $j \in T$ or deactivation if $j \in P$, $d_{ij} = 1$ indicates transcriptional activation by a member of T or activation by a member of P, and $d_{ij} = 0$ indicates the absence of an edge.

Graphs were constructed from the adjacency matrices used for the "gold standard" networks in the DREAM4 challenge in order to relate our inference performance to that achieved in the DREAM4 competition. The DREAM4 adjacency matrices were originally built from known biological networks to resemble a realistic transcription regulatory network. Edges in these networks could be organized into an adjacency matrix G with elements $g_{ij} \in \{0, 1\}$ indicating absence or presence of transcriptional regulation. Therefore, G readily describes $d(T, V)$, but not $d(P, R)$. The latter were designed by considering G as an indicator of edges in B, i.e. $g_{ij} = \mathrm{sgn}(b_{ij})$. As the desired adjacency matrix D has the same relationship to W (i.e.

$d_{ij} = \text{sgn}(w_{ij})$), we can construct D by considering the relationship from Eq (5) in the main text. Other constraints are a choice in the cardinality of P, as well as avoiding silent P regulation, e.g. phosphorylation cascades that terminates in a member of P rather than T.

First, nodes were assigned to the sets P, T or O. A node v_j was assigned to O if it has no transcriptional regulatory role, i.e. $\sum_i g_{ij} = 0$. Nodes in P require each of their regulatory effects to be mediated through a node in T. For this reason, nodes were iteratively assigned to P and T as follows: a randomly selected node v_j with at least one edge ($g_{ij} \neq 0$) is assigned to P if there exists another node v_k with the same target ($g_{ik} \neq 0$). In each iteration, any unassigned nodes v_k were assigned to T if they were the only potential TF regulator of a gene v_i (i.e. $\exists v_i : \sum_{v_j \notin P \cup O} g_{ij} = g_{ik} = 1$).

D is iteratively constructed, initially without cascades, with elements $d_{ij} \in \{0, 1\}$, based on G and the choices in P, T, and O. Elements for $v_j \in T$ were simply copied by assigning $d_{ij} := g_{ij}$. For $v_j \in P$ edges were iteratively added for each $g_{ij} = 1$ by randomly selecting a $v_k \in T$ that satisfies $d_{ik} = 1$ and setting $d_{kj} := 1$. Iteration for v_j is stopped when it holds $\forall i : \sum_k d_{ik} d_{kj} \geq g_{ij}$. Remaining entries of D are zero.

D is then modified to add phosphorylation cascades. If $\exists k \forall i : d_{ik} \leq d_{ij}$ then $d_{kj} := 1$, since this change does not increase the transcriptional influence of node v_j. Redundant edges were removed by setting $d_{ij} := d_{ij} - d_{ik}$ for each i. This process was repeated for any randomly selected $\{v_j, v_k\} \in P$ where it did not increase transcriptional influence of node v_j.

Lastly, entries in D were signed by setting $d_{ij} := -d_{ij}$ with a 50% probability. Adjacency matrix D indicates edge presence and mode of regulation and was given to GeneNetWeaverPhos, which assigned edge weights W. The weights were based on random decay rates (λ_i^+ and λ_i^- from Eq (9c) in the main text), numbers of competing negative and positive regulators, and other factors, such as the k_j of down-stream TF regulatory modules (1).

GeneNetWeaverPhos simulation

Simulation of the network in Fig 2 in the main text using GeneNetWeaverPhos is illustrated for wildtype and a knockout in Fig S2.

The initial concentrations for the wildtype simulation were estimated from assuming steady-state, which meant setting the differentials Eq (9a) and Eq (9b) in the main text to zero and approximating solutions for r and p. Although the resulting values were not the steady-state values observed from the simulation (for reasons of dependencies between terms), they were assumed to be close enough to the correct steady-state concentrations to converge during simulation. In all knockout examples tested, the same steady-state were reached whether the knockout initial conditions were the wildtype initial conditions or wildtype steady-state. In general we used the simulated steady-state values from wildtype as initial conditions for perturbation simulation. In knockout simulation the maximum transcription rate of the deleted gene is set to 1×10^{-5} (setting it to zero will result in NaN log fold-change values). The knockout simulation is terminated when it reached a new steady-state. Log fold-change values are then calculated as a measure of the mRNA concentrations of the knockout simulation relative to the wildtype simulation. PhosTF is only given these final log fold-change values and tries to reverse-engineer the protein interactions of the network.

Derivation of inference model

Perturbation experiment measurements were reported as $\log_2$ fold-change values. For gene i at time t the measurement is

$$x_i(t) = \log_2 \frac{x_i^{(\text{mut})}(t)}{x_i^{(\text{wt})}(t)}$$

$x_i^{(\text{mut})}(t)$ and $x_i^{(\text{wt})}(t)$ are absolute mRNA measurements for a mutant and wildtype. Equivalent values can be described for $y_i(t)$ and $a_i(t)$. We consider $y_i^{(\text{mut})}(t)$ and $y_i^{(\text{wt})}(t)$ to be the fractions of

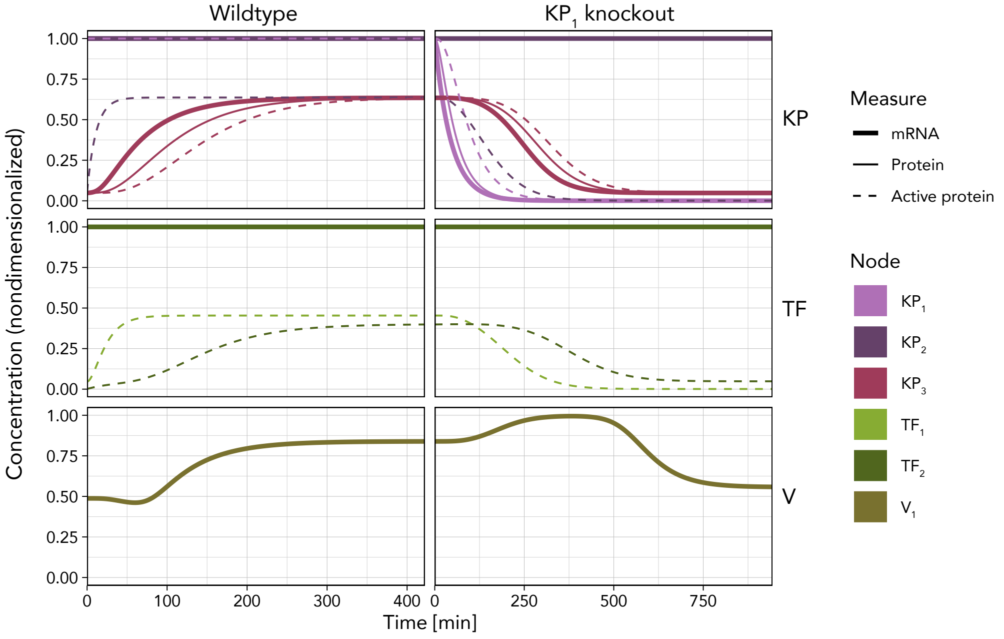

Fig S2. Simulation until steady state. Simulation of network in Fig 2 in the main text until convergence. Comparing wildtype and a mutant where P_1 has been knocked out. The values at convergence are seen in Fig 2D.

protein i which were actively eliciting their regulatory role, which gives the levels of active protein i, $a_i^{(\mathrm{mut})}(t) = y_i^{(\mathrm{mut})}(t) \cdot x_i^{(\mathrm{mut})}(t)$ and $a_i^{(\mathrm{wt})}(t) = y_i^{(\mathrm{wt})}(t) \cdot x_i^{(\mathrm{wt})}(t)$. It then follows that:

$$\begin{aligned} a_i(t) &= \log_2 \frac{y_i^{(\mathrm{mut})}(t) \cdot x_i^{(\mathrm{mut})}(t)}{y_i^{(\mathrm{wt})}(t) \cdot x_i^{(\mathrm{wt})}(t)} = \log_2 \frac{y_i^{(\mathrm{mut})}(t)}{y_i^{(\mathrm{wt})}(t)} + \log_2 \frac{x_i^{(\mathrm{mut})}(t)}{x_i^{(\mathrm{wt})}(t)} \\ &= x_i(t) + y_i(t) \end{aligned}$$

In vector notation the expressions for $\boldsymbol{x}(t)$ and $\boldsymbol{y}(t)$ were then inserted:

$$\begin{aligned} \boldsymbol{a}(t) &= \boldsymbol{x}(t) + \boldsymbol{y}(t) = (W_T + W_P)\boldsymbol{a}(t-1) + \boldsymbol{e}_x + \boldsymbol{e}_y \\ &= (W_T + W_P)^t \boldsymbol{a}(0) + \sum_{i=0}^{t-1} (W_T + W_P)^i (\boldsymbol{e}_x + \boldsymbol{e}_y) \\ &= W^t \boldsymbol{a}(0) + \sum_{i=0}^{t-1} W^i (\boldsymbol{e}_x + \boldsymbol{e}_y) \qquad \text{(If } P \cap T = \emptyset\text{)} \end{aligned}$$

Similar assumptions to those made for B in LLC were imposed on W: $\lim_{t\to\infty}(W_T + W_P)^t = 0$ and $\sum_{i=0}^{t-1}(W_T + W_P)^i$ converges for $t \to \infty$. This allows $\boldsymbol{a}(t)$ to converge and therefore $\boldsymbol{x}(t)$ and $\boldsymbol{y}(t)$ also converge, see Eq (4) in the main text. Time parameter t is omitted for values at

equilibrium:

$$\begin{aligned}
\boldsymbol{a} &= \boldsymbol{x} + \boldsymbol{y} = \boldsymbol{x} + W_P \boldsymbol{a} + \boldsymbol{e}_y \\
(I - W_P)\,\boldsymbol{a} &= \boldsymbol{x} + \boldsymbol{e}_y \\
\boldsymbol{a} &= (I - W_P)^{-1} \left(\boldsymbol{x} + \boldsymbol{e}_y\right) \\
\boldsymbol{x} &= W_T\,(I - W_P)^{-1} \left(\boldsymbol{x} + \boldsymbol{e}_y\right) + \boldsymbol{e}_x \\
&= W_T\,(I - W_P)^{-1}\,\boldsymbol{x} + \boldsymbol{e} \\
&= B\boldsymbol{x} + \boldsymbol{e} \quad , \quad B = W_T\,(I - W_P)^{-1}
\end{aligned}$$

Enhancing relative expression of genetically perturbed genes

Knocked out genes should ideally have expression rates of zero, generating log fold-change (logFC) values of $-\infty$. This was not the case in the perturbation data, where log fold-change values typically ranged from -2 to -5 (due to aspects of microarray technologies), although a few were unexpectedly observed to be positive. Overexpressed genes were expected to have higher expression rates than wildtype, and logFC values were typically in the range 0.5 to 3, although a few were close to 0 or slightly negative. For these reasons, measurements on the perturbed genes were artificially enhanced (Fig S3) (shifted by constant values) to ensure appropriate concentration parameters for these genes.

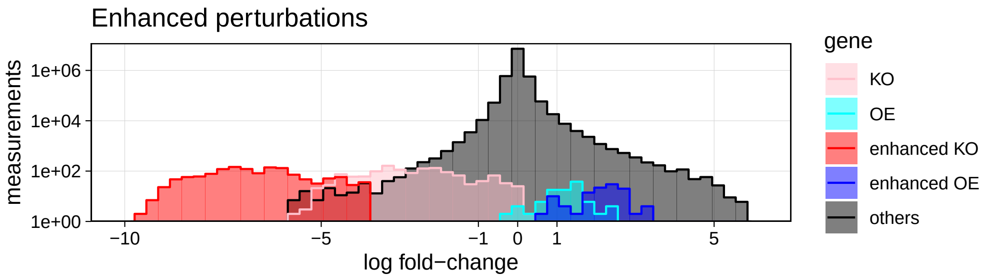

Fig S3. Enhancing perturbations Histogram of log fold-change values for the perturbation data. Gene measurements for mutated genes are shown before (”KO” and ”OE”) and after enhancement (”enhanced KO” and ”enhanced OE”). ”Others” refers to measurements of any gene that was not perturbed in a given experiment. The number of measurements for ”KO”, ”OE”, ”enhanced KO”, ”enhanced OE”, and ”others” are 1326, 100, 1343, 110, and 8578196 respectively.

Regularization strength on 4 type example

This section visualizes the impact on inference when choosing PhosTF regularization strength applied to the example in Fig 1, where 4 different edge combinations are present. Regularization strength is adjusted by setting the regularization factor λ in the loss function, see Eq (8) in the main text. Inferences using all relevant magnitudes are shown in Fig S4. The true network is fully recovered for $\lambda = 1$ and $\lambda = 0.1$, while $\lambda = 10$ is too strong a regularization, resulting in a missed (false negative) secondary regulation edge. $\lambda = 0.01$ and no regularization ($\lambda = 0$) was found to represent too little regularization, resulting in densely connected secondary regulatory networks with many false positives.

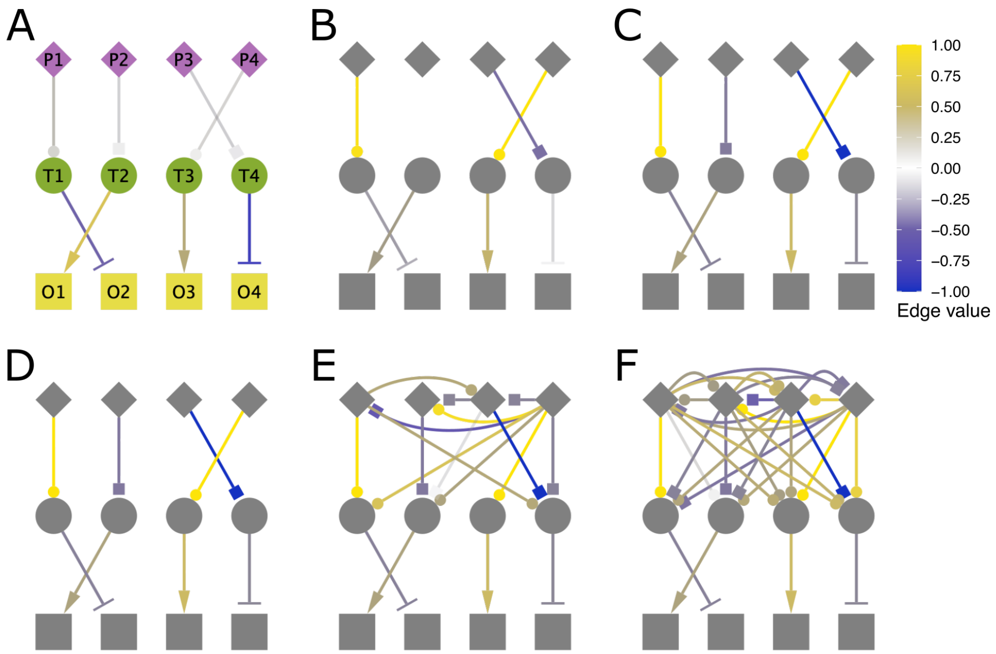

Fig S4. Regularization strength True network (A), and networks inferred from simulated log fold-change data (B-F). Regularization strength is determined by λ, which is set to 10 (B), 1 (C), 0.1 (D), 0.01 (E), and 0 (F).

References

1. Marbach D, Prill RJ, Schaffter T, Mattiussi C, Floreano D, Stolovitzky G. Revealing strengths and weaknesses of methods for gene network inference. Proceedings of the National Academy of Sciences of the United States of America. 2010;107(14):6286–6291. doi:10.1073/pnas.0913357107.

2. Von Dassow G, Meir E, Munro EM, Odell GM. Formulation of a model of the segment polarity network as a system of first-order ordinary differential equations using Ingeneue Supplementary Information for ”The Segment Polarity Network is a Robust Developmental Module”; 2000. Available from: `http://rusty.fhl.washington.edu/ingeneue/downloads/NatureSupplement.pdf`.
